# A Reliable Parameter to Standardize the Scoring of Stem Cell Spheres

**DOI:** 10.1371/journal.pone.0127348

**Published:** 2015-05-14

**Authors:** Xiaochen Zhou, Gongxian Wang, Yi Sun

**Affiliations:** 1 Department of Urology, The First Affiliated Hospital of Nanchang University, Nanchang, Jiangxi, P.R. China; 2 Division of Radiation and Cancer Biology, Department of Radiation Oncology, University of Michigan, Ann Arbor, Michigan, United States of America; 3 Institute of Translational Medicine, Zhejiang University School of Medicine, Hangzhou, Zhejiang, P. R. China; 4 Collaborative Innovation Center for Diagnosis and Treatment of Infectious Diseases, Zhejiang University, Hangzhou, China; Second University of Naples, ITALY

## Abstract

Sphere formation assay is widely used in selection and enrichment of normal stem cells or cancer stem cells (CSCs), also known as tumor initiating cells (TICs), based on their ability to grow in serum-free suspension culture for clonal proliferation. However, there is no standardized parameter to accurately score the spheres, which should be reflected by both the number and size of the spheres. Here we define a novel parameter, designated as Standardized Sphere Score (SSS), which is expressed by the total volume of selected spheres divided by the number of cells initially plated. SSS was validated in quantification of both tumor spheres from cancer cell lines and embryonic bodies (EB) from mouse embryonic stem cells with high sensitivity and reproducibility.

## Introduction

Sphere formation assay, which was initially designed to isolate and culture neural stem cells more than 20 years ago [1], has now been widely used for assessment of stemness and enrichment of CSCs or TICs. CSCs/TICs are a small group of tumor cells with stem cell properties including self-renewal, differentiation, proliferation, and multi-drug resistance. These cells are characterized as being able to form free floating spherical three-dimensional structures (tumor spheres, TS) in non-adherent condition and serum-free media supplemented with growth factors and other required nutrients. CSCs are capable of self-renewing, and serial passaging of TS allows for selection of self-renewing CSCs [2]. Sphere formation assay has been successfully applied for the generation and maintenance of CSCs/TICs with higher tumorigenicity [3] in various human cancers including carcinomas of breast [4], colon-rectum [5], lung [6], head and neck [7], and bone [8, 9].

Though in theory, each sphere is derived from a single cell and plating single cell per well remains to be the golden standard to characterize stem cell potential, it is important to note that under one cell per well condition, the rate of TS formation is extremely low, likely due to the lack of autocrine and/or paracrine signals released by co-cultured cells into the medium [2]. Thus, TS formation assay was normally performed as multiple–cell plating at clonal density (0.2 to 20 cells per μl) [10] for proper clonal growth. Likewise, since formation of a sizable tumor mass *in vivo* by injection of single unselected tumor cell is a very rare event [11], the tumorigenesis assay was usually conducted by injection of “a group” of selected or unselected tumor cells. Therefore, TS formation assay via multiple-cell plating is popularized for studies of CSC proliferation, and might be more appropriate in mimicking the in vivo scenario of tumorigenesis.

In sphere formation assay, stem cell frequency is calculated based on the number of spheres, which is not accurate, since besides neural stem cells (NSCs) or mammary stem cells (MaSCs), FACS selected progenitor cells derived from NSCs or MaSCs could also form neurospheres or mammospheres, respectively [12]. Overestimation on stem cell frequency could happen when the calculation is solely based on number of spheres [13].

With regard to the size of spheres, it is generally believed that stem cells give rise to larger spheres while progenitor cells which loss the ability of self-renewal give rise to smaller spheres [2]. The size of spheres obviously reflects the proliferation of sphere forming cells. Currently, the number of TS or TS forming efficiency is the most commonly used parameter to score TS generated by either multiple-cell plating or single-cell plating approach [14]. Other parameters include mean diameter of spheres [15], representative pictures and morphological description [16], relative area occupied by spheres [17], and simply with or without TS formation [18]. Currently there is no parameter to accurately measure TS formation ability, which takes both the number and size of spheres into account.

Here we describe a novel parameter to assess TS formation capacity designated as Standardized Sphere Score (SSS), a single quantifiable parameter to reflect both the number and size of spheres. We also provide proof-of-concept evidence, showing that SSS objectively reflects the sphere forming ability or proliferation capacity of sphere forming cells with high sensitivity and reproducibility.

## Materials and Methods

### Cell lines

HCT116 (colorectal cancer), H1299 (lung cancer) and MCF7 (breast cancer) cell lines were obtained from ATCC. Mouse embryonic stem cells were derived as previously described [19].

### Drugs and treatment

Canertinib (CI-1033, Pfizer Pharmaceuticals) and Erlotinib (Selleck Chemicals) were kindly provided by Dr. Mukesh Nyati at University of Michigan. Perifosine (Sigma), MK2206 (Selleck Chemicals) and BEZ235 (Cayman Chemical) were kindly provided by Dr. Alnawaz Rehemtulla at University of Michigan. For drug treatment, we used CI-1033 at 2 μM; Erlotinib at 3 μM, 6 μM and 10 μM, MK2206 at 0.25 μM, 0.5 μM and 1 μM; Perifosine at 1 μM, 3 μM and 5 μM; and BEZ235 at 25 nM, 125 nM and 250 nM. Above drugs were used to treat H1299 cells in tumor sphere formation assay for 4 days. Individualized drug concentrations were set according to cellular response to each drug for a common period of time.

### Sphere formation assay

For TS formation assay, tumor cells were maintained as monolayer culture before being harvested as single cell suspension by enzymatic/mechanic disassociation, washed in 1×PBS twice, and filtered through 40 μm cell strainer. Cells were than counted and plated at clonal density (≤ 1 cell per μL medium) in TS medium (DMEM/F12 (Gibco) supplemented with 20 ng/ml human recombinant epidermal growth factor (EGF, Gibco), 10ng/ml human recombinant basic fibroblast growth factor (FGF-2, Sigma), 0.4% BSA (Fraction V, Fisher), 5mM HEPES (Gibco) and 1× Penicillin-Streptomycin (Gibco)) in 24-well ultra-low attachment (ULA) plates (Corning). We allowed TS formation for up to 16 days, performing microscopic assessment every 4 days. For embryonic body (EB) formation assay, mouse embryonic stem cells (mESCs) were maintained in DMEM (Gibco) supplemented with 15% FBS (Gibco) and 1 ng/mL recombinant human leukemia inhibitory factor (LIF, Gibco), before being harvested as single cell suspension by enzymatic/mechanic disassociation, and filtered through 40 μm cell strainer. Cells were than counted and plated at clonal density in DMEM supplemented with 15% FBS (unless otherwise indicated) and 1 ng/mL LIF in 24-well ULA plates. We allowed EB formation for up to 8 days, performing microscopic assessment every 4 days.

### Measurement and calculation of Standard Sphere Score

SSS can be calculated by manual measurement of diameter of spheres under light microscope assisted with computer-based imaging software with the function of taking live measurements (NIS Elements BR), or automatically by commercially available cytometers (Celigo S Cell Cytometer).

### H&E staining of tumor spheres

H1299 cells were cultured in TS forming condition for 8 days, before being harvested. Intact H1299 TS were then fixed in 4% paraformaldehyde, dehydrated and paraffin embedded. The 5μm-thick sections were obtained followed by standard H&E staining.

### Statistical analysis

All experiments with drug treatment were repeated at least three times, or otherwise indicated. Graphs were plotted in Excel (Microsoft) or GraphPad (Prism). Data were presented as Mean ± SEM.

## Results and Discussion

### Characteristics of tumor spheres

**Fig 1:**
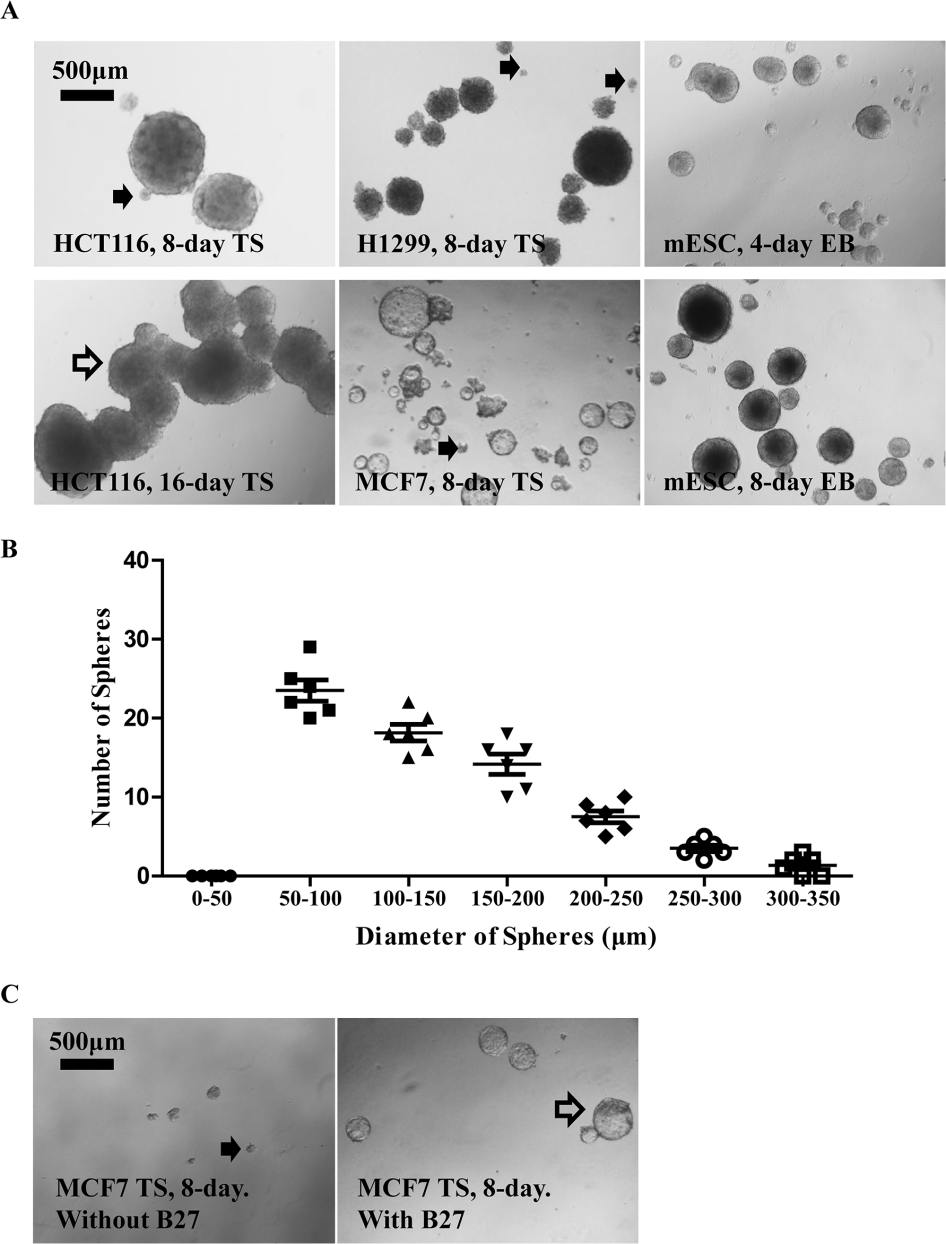
General characteristics of tumor spheres formed from multiple cell lines. **A**. Significant heterogeneity existed in the size of TS as shown by representative pictures of TS from H1299 and MCF7 cultured in TS medium for 8 days. Counting of smaller TS (solid arrow) was sometimes prone to subjectiveness. TS derived from certain cell lines (HCT116) might have strong tendency to fuse (hollow arrow). **B.** “size of spheres” for H1299 TS showed a skewed distribution with smaller spheres account for the majority of population. H1299 cells were plated in 24-well ULA plate (2000 per well in 2ml of TS medium) to allow TS formation for 4 days before microscopic assessment. The results were presented as Mean ± SEM from 6 replicates. **C**. Some tumor cell lines formed typical 3-dimensional spherical structures with sharp edges (A), while others were not, or require special supplement (hollow arrow).

Sphere formation assay has been successfully used for the isolation and enrichment of CSC in lung cancer, colorectal cancer and breast cancer. Pulmospheres, colospheres and mammospheres generated in condition medium for tumor sphere formation (TS medium) expressed higher level of CD133/CD44 [5, 20], CD133 [21], or enriched CD44+/CD24- subpopulation [4], respectively, indicating their cancer stem cell phenotype. We therefore cultured lung H1299, colon HCT116 and breast MCF7 cancer cells in TS medium for demonstration of general characteristics of tumor spheres culture which is commonly used in the study of CSCs. Consistent with previous observation [2], we found a significant heterogeneity in the size of TS from a variety of human cancer cell lines (Fig 1A). The size of TS in these cancer cell lines presented a skewed distribution with smaller spheres accounting for the major of population (Fig 1B), inconsistent with a Gaussian distribution reported in one study [22]. Although a TS typically ranges from 50 to 250 μm in diameter, the arbitrate setting on sphere size cut-off introduces inconsistency among different studies, not to mention the fact that smaller TS are more difficult to define due to their irregular morphology (Fig 1A, solid arrow). Mouse embryonic stem cells (mESCs) (Fig 1A) and some tumor cell lines are able to form typical 3-dimensional spherical structures with sharp round edges, while others are not. Still others require additional supplement in TS medium (e.g B27 supplement [23]) for effective formation of spherical TS (Fig 1C). The tendency of sphere-sphere fusion also varies among TS generated from different cancer cell lines (Fig 1A, hollow arrow). All these variations in morphological characteristics of spheres, along with individual’s subjectivity to rule in or rule out a given small sphere, make it a challenging task for precise sphere counting.

### Limitations of current parameters in sphere scoring

Limitations of current parameters to score sphere formation assay include 1) to describe the presence of spheres with representative pictures, which is certainly not a quantifying assessment; 2) to count the number of sphere to reflect TS forming frequency, but lack of evaluation of the TS size, an index of proliferation as well as stem cell potential [2]; 3) to measure the mean diameter of TS (a first power parameter), or relative area occupied by TS (a second power parameter) [15, 17]. It can, to some extent, reflect both TS frequency and proliferation potential. However, mathematically, the size of TS should be most accurately represented by the volume of a sphere (a third power parameter).

### Definition of Standardized Sphere Score (SSS)

To precisely assess the potential and proliferation of CSC in TS formation assay, we need a parameter that takes both number and volume of TS into account. We therefore proposed a novel parameter as standardized sphere score, which is expressed by the sum of volume of all spheres counted, divided by the total number of cells initially plated. SSS can be calculated as $SSS=\frac{V_{sum}}{N}=\frac{\pi }{6N}\sum_{i=1}^{n}D_{i}{}^{3}$ (for abbreviations, V_sum_: total volume of all spheres; N: total number of cells initially plated; n: total number of spheres; D_i_: diameter of a sphere).

**Fig 2:**
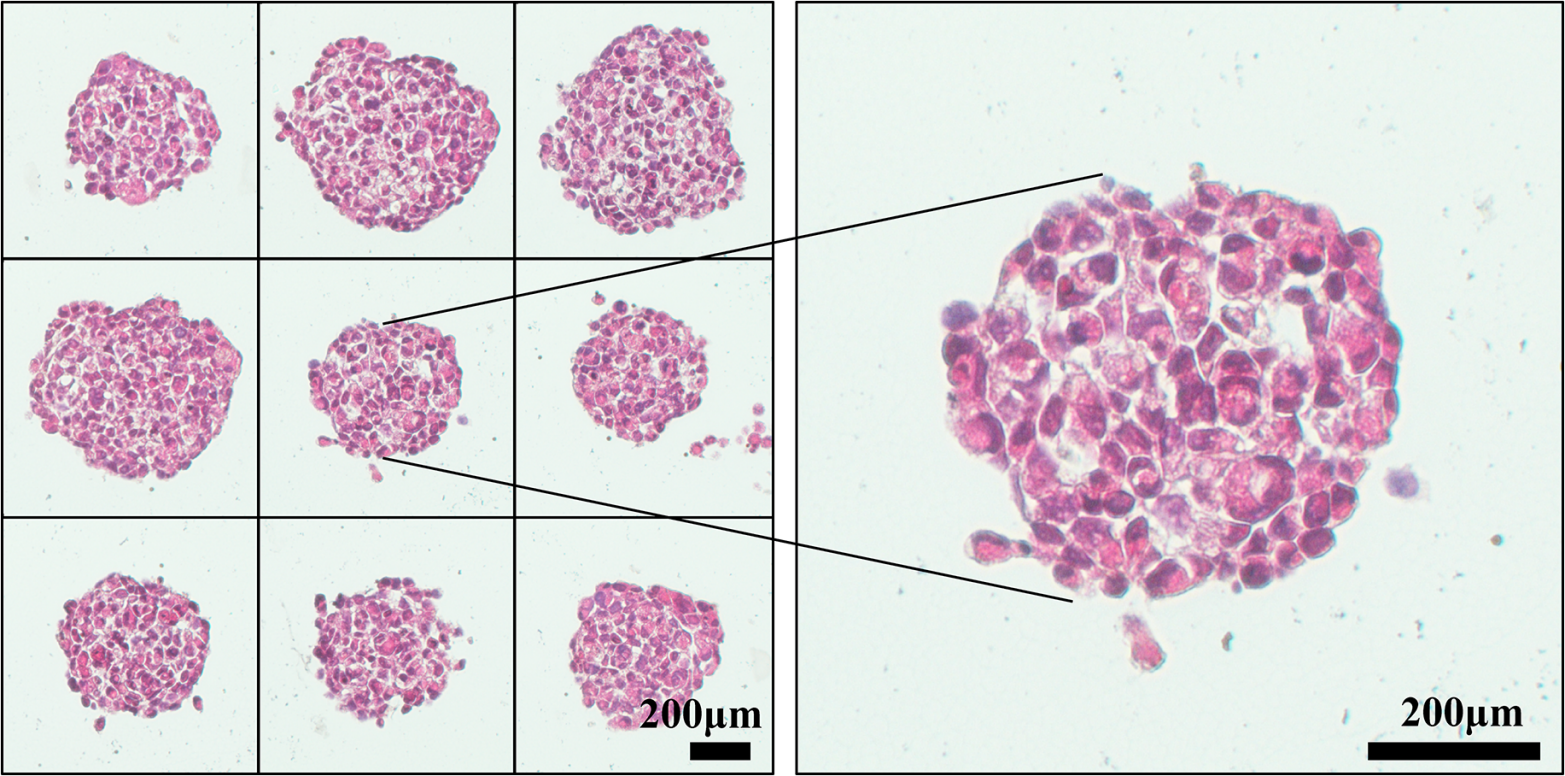
H&E Staining of H1299 TS. H1299 cells were cultured in TS forming condition for 8 days, before being harvested, fixed, paraffin embedded, sectioned, followed by H&E staining to show how single tumor cells were packed in TS.

**Fig 3:**
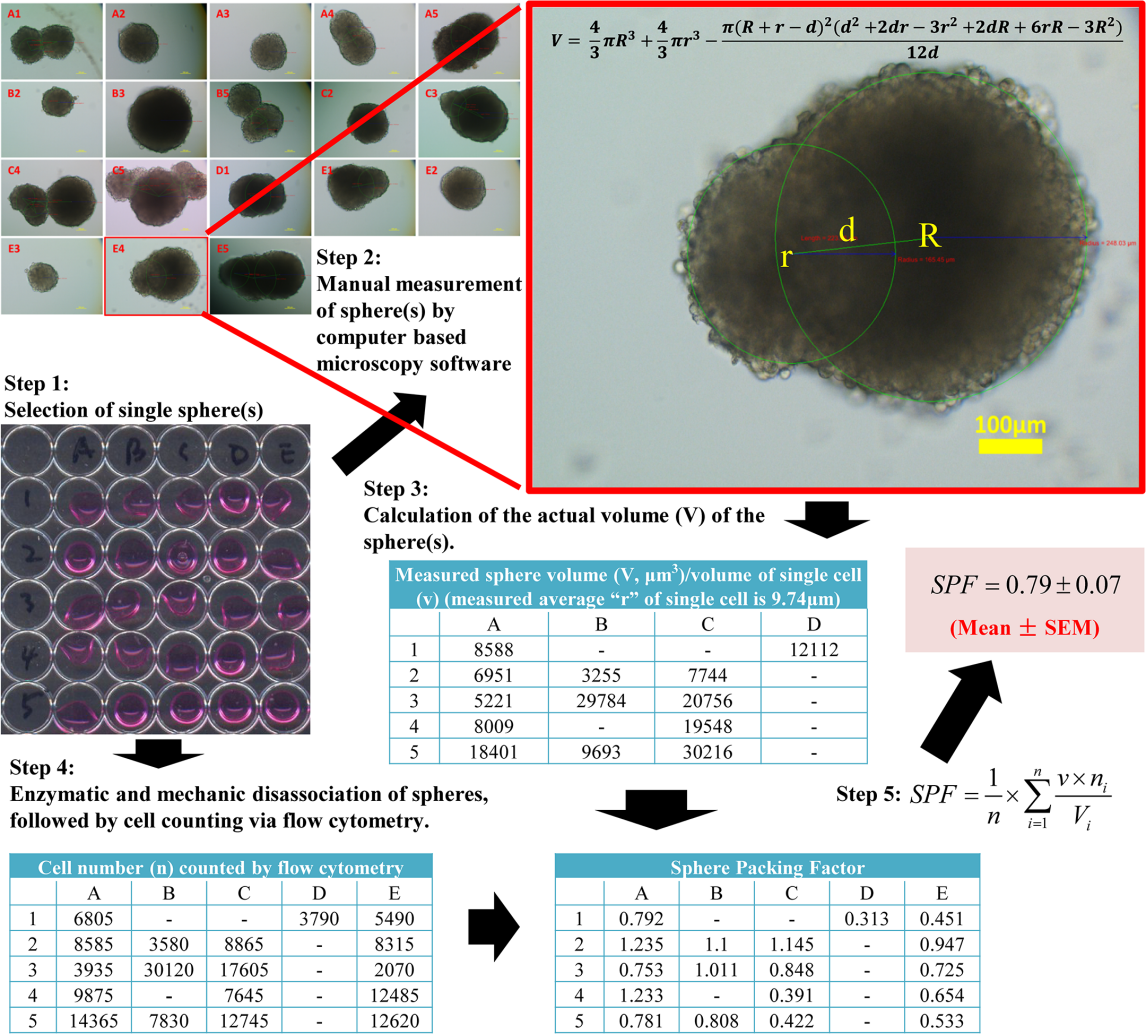
A general protocol for SPF calculation using H1299 TS. Step 1: Spheres were harvested and seeded individually in each well. Step 2: The diameter of spheres was measured by computer-based imaging software, and documented. Note that the three labels in red are software generated measurement of distance between centers of two spheres (223.08 μm), radius of left sphere (165.45 μm) and radius of right sphere (248.03 μm), respectively (from left to right). Step 3: The volume of each sphere was calculated based on measured diameter obtained from step 2. Step 4: Spheres were disassociated enzymatically and mechanically to make single cell suspension, followed by precise cell counting by a cytometry. Meanwhile, the diameter of single floating cells from spheres was measured. Step 5: SPF was calculated by an indicated formula.

In order to accurately use SSS to quantify sphere formation, we borrowed the term “atomic packing factor (APF)” used in crystallography [24] to introduce a terminology, Sphere Packing Factor (SPF) as an index of “tightness” of a sphere. Assuming that spheres of varying sizes derived from a given cell line are “packed” identically in the same culturing condition (sharing the same SPF), the volume of spheres or SSS will be directly correlated with number of cells in spheres, which in turn, is directly related to the proliferative status of sphere-forming cells. When assessing sphere formation of a given line in different culturing conditions (i.e., with or without a drug treatment), the possibility that culturing conditions may alter SPF should be considered while using SSS for quantification of spheres. The idea of SPF has been previously adapted in multicellular tumor spheroids to reflect extracellular matrix distribution and cell to cell or cell to matrix interactions [25]. By definition, SPF is defined as “number of single cells in the sphere” times “average volume of a single cell” divided by “measured volume of the sphere”. SPF can be calculated as $SPF=\frac{1}{n}\times \sum_{i=1}^{n}\frac{v\times m_{i}}{V_{i}}$ (for abbreviations, n: total number of spheres included in the calculation of SPF; v: volume of a free floating single cell; m_i_: number of cells within a sphere; V_i_: volume of a sphere). Based on the definition, the relationship between SSS and SPF can be expressed as $SSS=\frac{v}{N\times SPF^{}}\times m_{sum}$. From the above equation, it is evident that v (volume of a free floating single cell), the nature of a cell line, is a constant; N (total number of cells initially plated) is a user-defined constant; therefore, if SPF is also a constant, SSS is then a direct read-out of m_sum_ which represents the total number of cells in all spheres. We can also see that SPF is a factor with a decimal value from 0 to 1 without any unit. The “tightness” of H1299 TS, which reflects the cell-cell association within a sphere is shown in Fig 2. We also used H1299 TS as an example to illustrate how to measure and calculate SPF (Fig 3).

### Validation for SSS in quantification of sphere formation assay

Based upon cancer stem cell theory [26], the percentage of CSC/TIC, a subset of cancer cells with stem cell property out of the total cell population, or the stem cell potential, should be constant for a given cell line. In other words, the percentage of CSC/TIC, or stem cell potential should not be changed regardless of how many cells are seeded initially. Thus, in sphere formation assay with a series of dilution (plating a serially decreasing number of cells), if a straight line crossing the origin can be reached by plotting SSS×N (SSS times number of cells plated, being the y-axis) against N (number of cells plated, being the x-axis), it can be concluded that SSS objectively reflects the proliferation of those sphere forming cells, or stem cell potential. To be sure, we plotted number of spheres (n) against N and expected to see a linear relationship between the two, based on cancer stem cell theory [27] and a recognized assumption that number of spheres reflects stem cell potential of a given population [2]. The straight line obtained from plotting n against N would further confirm the above acknowledged theories; meanwhile, it could serve as a proof that we plated accurate number of cells as planned in the following assays.

**Fig 4:**
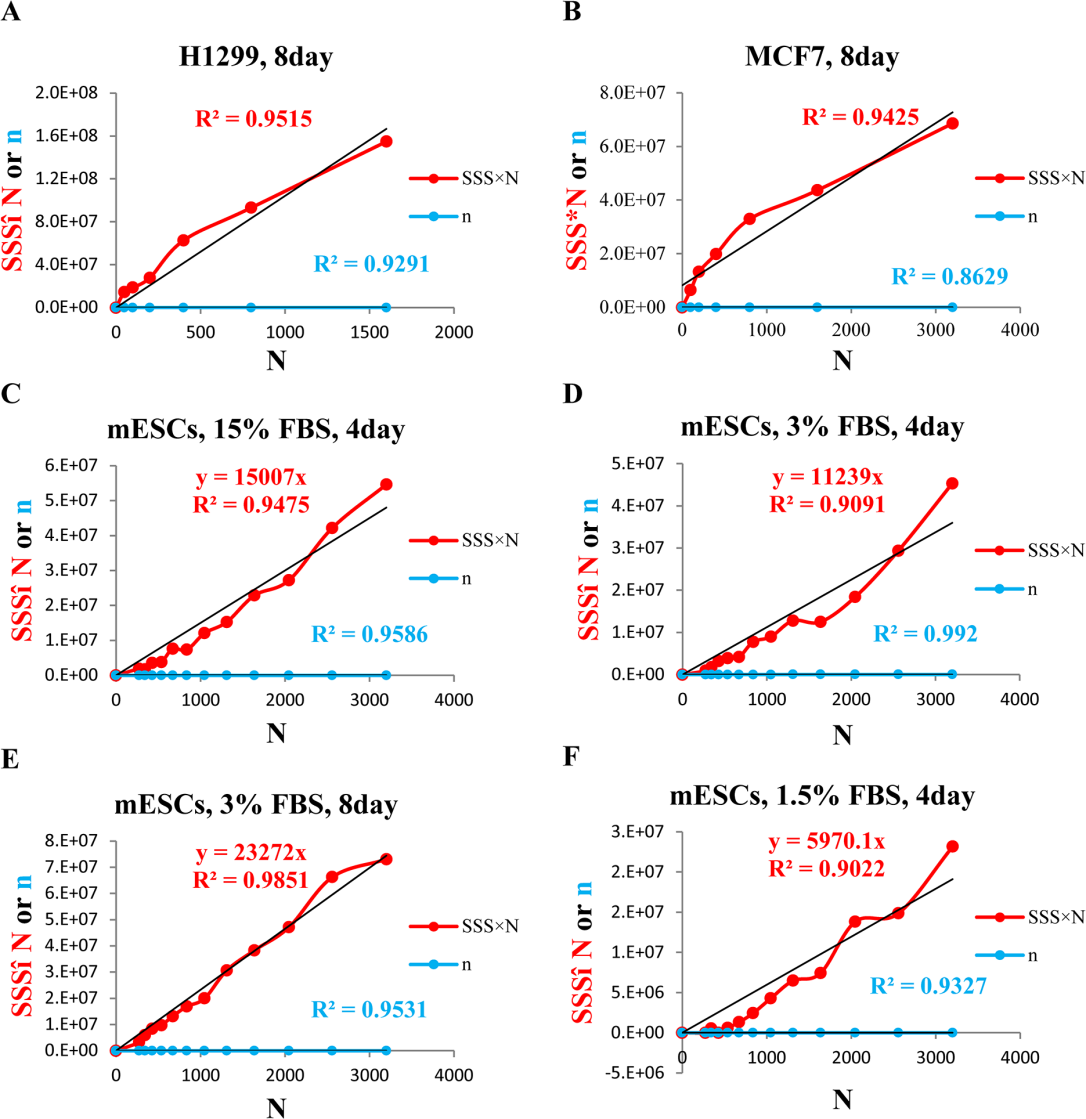
Standardized Sphere Score (SSS) linearity validation. **A.** H1299 cells (plating cell number was 1600 × 0.5^*i*^,*i* = 0,1,2…5 per well) were plated in TS medium in 24-well ULA plates for 8 days to allow for formation of TS. Number of spheres (n) and SSS were then measured and calculated. The results were presented with SSS×N and n as the Y-axis, and N as the X-axis. **B.** MCF7 cells (plating cell number was 3200 × 0.5^*i*^,*i* = 0,1,2…5, per well) were plated in TS medium supplemented with 1× B27 in 24-well ULA plates for 8 days to allow for formation of TS. Number of spheres (n) and SSS were then measured and calculated. The results were presented with SSS×N and n as the Y-axis, and N as the X-axis. **C-F**. mESC (plating cell number was 4000*0.8^*i*^,*i* = 0,1,2…11, per well) were plated in 24-well ULA plates in DMEM supplemented with LIF and, respectively, with 15% FBS (C), 3% FBS (D, E), and 1.5% FBS (F) for up to 8 days, to allow the formation of EB. Number of EBs (n) and SSS were similarly obtained. Since the numerical value of n is much smaller than that of SSS×N, the plots of n against N appeared to be very close to the X-axis. Each experiment was repeated at least 3 times.

We first tested our hypothesis in H1299, an NSCLC cell line which formed typical TS with ideal spherical shape and sharp edges. Indeed, plotting n against N generated a straight line (fixed intercept at origin) with a correlation co-efficiency (R^2^) of 0.93, confirming the theory that stem cell potential is a constant that is irrelevant with number of cells plated (Fig 4A). It also proved that we plated cells accurately. Based on the above, another perfect straight line (fixed intercept at origin) with R^2^ of 0.95 generated from plotting of SSS×N against N indicated that SSS objectively reflects stem cell potential (Fig 4A). Similar result (R^2^ of 0.94 generated from plotting of SSS×N against N) obtained from another cancer cell line (MCF7), confirmed that the linearity of SSS was not cell line dependent (Fig 4B).

We next tested SSS using mouse embryonic stem cells (mESCs). In non-adherent culturing condition supplemented with LIF to prevent stem cell differentiation, mESCs are capable of forming embryonic bodies (EBs) with perfect spherical structure. At day 4, although the rate of proliferation is directly correlated with the concentrations of FBS as indicated by slopes of trend lines (fixed intercept at origin) generated from plotting of SSS×N against N in 1.5% FBS (*y* = 5970.1*x*), 3% FBS (*y* = 11239*x*) and 15%FBS (*y* = 15007*x*), these trend lines were perfectly linear as indicated by their respective R^2^ of greater than 0.9 regardless of amount of serum used (Fig 4C, 4D and 4F). We also tried to test the linearity of SSS at 8 day time point. However, EBs in 15% FBS developed necrotic center whereas EBs in 1.5% FBS no longer maintained ideal morphology (data not shown), they were therefore excluded from the study. From the results of EBs cultured in 3% FBS for 8 days, we observed again, a perfect linear relationship between SSS×N and N, further supporting the universal adaptability of SSS in reflecting stem cell potential (Fig 4E). Trend lines generated from plotting n against N were all linear as indicated by their respective R^2^ of greater than 0.90, which served as a control for cell plating here.

These results indicated that for both normal stem cell and CSC, SSS could objectively reflect the proliferation status of those sphere-forming cells, or stem cell potential.

### Application of SSS in scoring of sphere forming assay after drug treatment

**Fig 5:**
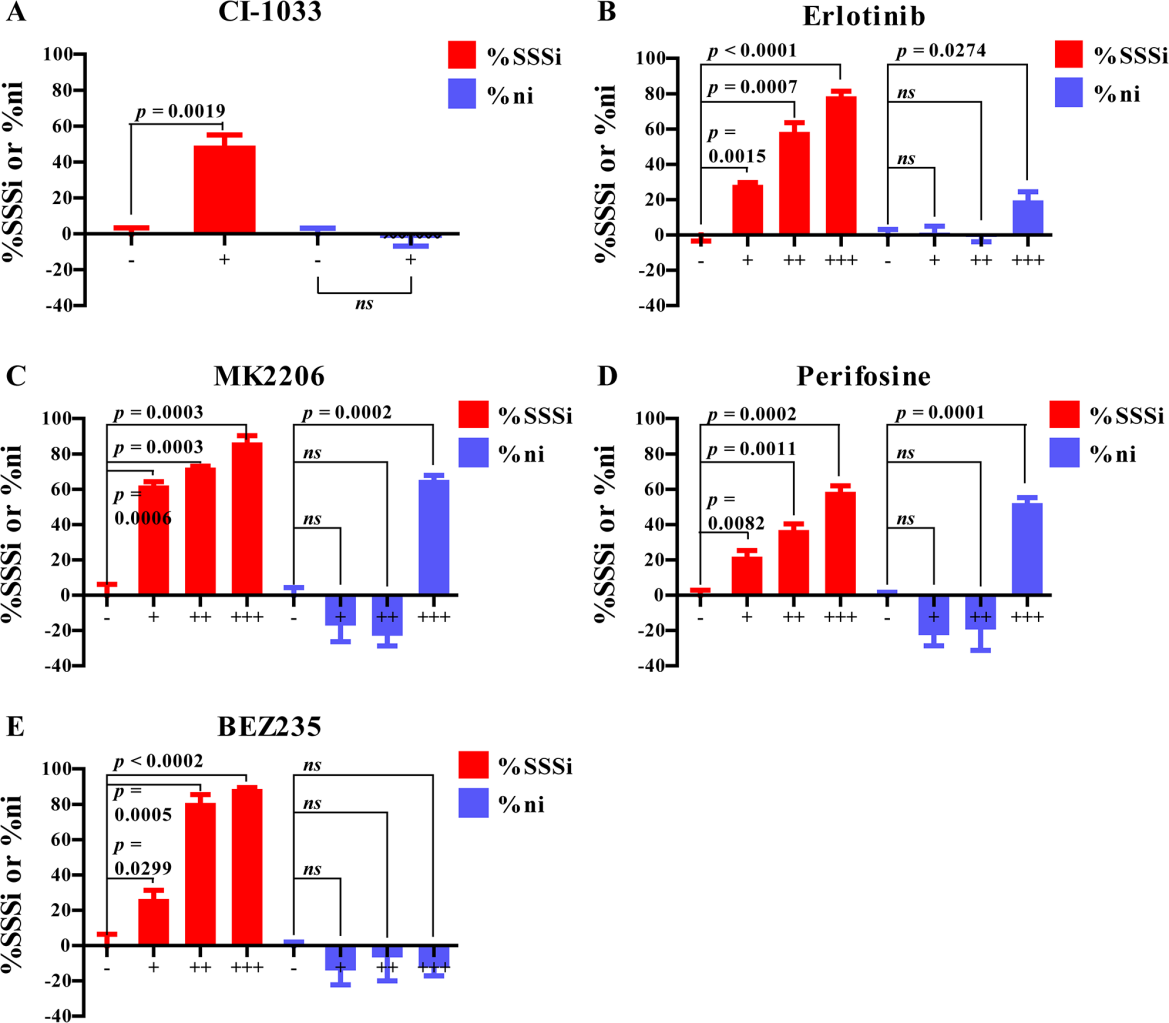
Dose dependent inhibition of sphere formation by anticancer drugs. To compare two sphere scoring parameters of the SSS vs. “number of spheres”, H1299 (2000 cells per well) were plated in 24-well ULA plates in TS medium supplemented with indicated drugs or vehicle for 4 days. %SSS_i_ and %n_i_ were then calculated. Concentration of drugs were as the following: (A) CI-1033: + 2 μM; (B) Erlotinib: + 3 μM, ++ 6 μM, +++ 10 μM; (C) MK2206: + 0.25 μM, ++ 0.5 μM, +++ 1 μM; (D) Perifosine: + 1 μM, ++ 3 μM, +++ 5 μM; and (E) BEZ235: + 25 nM, ++ 125 nM, +++ 250 nM. The results were presented as Mean ± SEM.

We next determined how accurate or sensitive SSS can be in assessing sphere formation. We tested several known drugs with anti-tumor activity in TS formation assay and scored the results by SSS in comparison with conventional parameters, after confirming that drugs did not cause a statistically significant change in SPF. To intuitively show the inhibitory effect of selected drugs on TS formation, we defined two parameters termed as %SSS_i_ and %n_i_, and calculated as $\%SSS_{i}=(1-\frac{SSS^{Drug}}{SSS^{Vehicle}})\times 100\%$ and $\%n_{i}=(1-\frac{n^{Drug}}{n^{Vehicle}})\times 100\%$, respectively (for abbreviations, SSS^Vehicle^: Standardized Sphere Score of cells treated with vehicle; SSS^Drug^: Standardized Sphere Score of cells treated with selected drug; n^Vehicle^: number of spheres from cells treated with vehicle; n^Drug^: number of spheres from cells treated with selected drug). As explained by the equations, the higher value of %SSS_i_ or %n_i_, the stronger inhibitory effect of the drug on SSS or number of spheres (n), respectively. Negative value of %SSS_i_ or %n_i_ indicates that the drug causes an increase in SSS or n respectively. CI-1033 is a highly selective irreversible tyrosine kinase inhibitor (TKI) against ErbB family members, which showed both *in vitro* [28] and *in vivo* [29] antitumor activity against a number of human cancers including non-small-cell lung cancer (NSCLC). In agreement with a previous report that CI-1033 only reduced the “size of TS” but not “number of TS” [30], we observed a dose dependent decrease in SSS, with no significant (ns) changes in number of spheres (Fig 5A). We subsequently tested several other drugs with anti-tumor activity, including Erlotinib (reversible TKI targeting EGFR) [31], MK2206 (allosteric inhibitor of Akt1/2/3) [32], Perifosine (a PI3K/AKT inhibitor) [33], and BEZ235 (dual ATP-competitive PI3K/mTOR inhibitor) [34]. In every single case, a dose dependent increase in value of %SSS_i_ was observed, as evidenced by decrease in p value, but not the value of %n_i_ (Fig 5B–5E), indicating that SSS could more objectively and sensitively reflect changes in TS formation.

### Pros and cons of SSS in quantification of sphere formation assay

The pros and cons of SSS in sphere scoring are listed in Table 1. The most significant advantage is its high sensitivity to detect the effect of drugs in a dose-dependent manner, while number of spheres fails to exhibit a similar patter as demonstrated in Fig 5. Another advantage is that SSS could be more effective in minimizing the counting errors due to misjudgment on spheres, particularly those smaller spheres with possibly less ideal morphology, which may contribute less to overall proliferation status or stem cell potential. Inclusion or exclusion of a small sphere could greatly affect the score with other parameters such as “number of spheres/sphere forming efficiency” or “mean of diameter of spheres”. However, it has little effect while using SSS to score. For example, assuming we have a pool of 50 spheres generated from plating N×cells, among which 49 spheres have diameter of 200 μm, whereas one sphere has diameter of 50 μm. Inclusion or exclusion of this small sphere will introduce a Mean Percentage Error (MPE) of 2.0% using either “number of spheres” (50 vs. 49) or “sphere forming efficiency” (50/N vs. 49/N); and a MPE of 1.52% using “mean of diameter of spheres” (197 μm vs. 200 μm). However, the MPE when using SSS will be 0.03% (392,125,000/50 vs. 392,000,000/50) which is much smaller than the MPE when using other parameters. The advantage of SSS in terms of MPE would be much more significant, if those small spheres that are prone to counting error or subjectiveness account for a major portion. Given the common features in TS pool, including heterogeneity in size and error-prone counting of small spheres which may be a major portion of the population, SSS appears to be the most accurate parameter.

**Table 1:** Pros and cons of SSS in assessing sphere formation assay.

**Pros**	**Sensitivity**	More sensitive than other parameters in detecting changes in sphere forming ability
	**Accuracy**	Minimize the counting error from identifying and measuring smaller spheres
	**Adaptability**	no need to harvest spheres
		assessment at different time points
		subsequent functional experiments on intact spheres.
**Cons**	**Morphology**	require typical spherical structure
		may not be applicable when significant fusion of spheres is present
	**Time consuming**	manual counting of a 24-well plate with each well containing 50~100 tumor spheres typically requires 30~60 minutes.

On the other hand, SSS has its limitations in assessing sphere formation assay. First of all, the size of spheres needs to be measured with acceptable accuracy. This requires spheres having typical spherical shape, while not having significant fusion among them. Secondly, measurement and calculation of SSS will be more time consuming than simply counting the numbers. To obtain SSS of all wells in a 24-well plate with each well containing around 50–100 spheres, it typically takes 30–60 minutes by manual counting with the assistance of computer based imaging software (for example, NIS Element BR) for measurement in live microscopic view.

## Conclusions

Here we introduce this new parameter, Standardized Sphere Score (SSS) to standardize the sphere scoring system. SSS is sensitive, reliable, and accurate to score spheres with manageable errors. The scoring can be achieved by manual counting using a microscope coupled with imaging software with live-measuring function, or by automatic counting systems that are commercially available (e.g. Celigo S Cell Cytometer).
